# Charge Trap States of SiC Power TrenchMOS Transistor under Repetitive Unclamped Inductive Switching Stress

**DOI:** 10.3390/ma15228230

**Published:** 2022-11-19

**Authors:** Juraj Marek, Jozef Kozarik, Michal Minarik, Aleš Chvála, Matej Matus, Martin Donoval, Lubica Stuchlikova, Martin Weis

**Affiliations:** 1 Institute of Electronics and Photonics, Slovak University of Technology in Bratislava, 812 19 Bratislava, Slovakia; 2 NanoDesign, s.r.o, Drotárska 19a, 811 04 Bratislava, Slovakia

**Keywords:** charge trap states, reliability, degradation, SiC MOSFET, TrenchMOSFET, repetitive UIS

## Abstract

Silicon carbide (SiC) has been envisioned as an almost ideal material for power electronic devices; however, device reliability is still a great challenge. Here we investigate the reliability of commercial 1.2-kV 4H-SiC MOSFETs under repetitive unclamped inductive switching (UIS). The stress invoked degradation of the device characteristics, including the output and transfer characteristics, drain leakage current, and capacitance characteristics. Besides the shift of steady-state electrical characteristics, a significant change in switching times points out the charge trapping phenomenon. Transient capacitance spectroscopy was applied to investigate charge traps in the virgin device as well as after UIS stress. The intrinsic traps due to metal impurities or $Z_{1,2}$ transitions were recognized in the virgin device. The UIS stress caused suppression of the second stage of the $Z_{1,2}\;$ transition, and only the first stage, $Z_{1}^{0}$, was observed. Hence, the UIS stress is causing the reduction of multiple charging of carbon vacancies in SiC-based devices.

## 1. Introduction

Due to their superior properties, silicon carbide-based (SiC) devices have become the number one choice for power electronic conversion semiconductor (PECS) applications, where they operate under high power, high temperature, and high switching frequency conditions [1,2]. These applications typically employ a high quantity of large-area dies per system while demanding high system-level reliability under aggressive electrical and environmental operating conditions. Significant progress in recent years in the development of SiC transistors and, above all, in their reliability have contributed to the fact that SiC-based devices have become a viable alternative to silicon-based power devices [3]. Nevertheless, before SiC can completely replace silicon (Si) counterparts, robustness and reliability remain the main issue [4,5] for the devices under several extreme operational conditions, such as overcurrent, overtemperature, short circuit, and unclamped inductive switching (UIS) [6]. For example, sudden high currents and voltage pulses may occur during start-up events or load changes of electric motors. Even with a good inverter or power supply design, high-voltage peaks may occur due to inevitable parasitic inductances. As a result, voltage spikes can easily reach the value of the breakdown voltage of the switch, leading to an avalanche breakdown operation for a short time. This imposed stress might trigger the drift of electrical parameters that can limit the devices’ operating range and lifetime. Even though the degradation of device electrical parameters is well studied, the relation to material defects is still unclear and represents a gap in knowledge of SiC-based devices.

Therefore, in our analysis, we focused on the impact of repetitive unclamped inductive switching (UIS) on the stability of electrical parameters of commercial 1.2-kV 4H-SiC MOSFETs with an asymmetric channel configuration proposed by R. Siemieniec [7]. The stress-induced shift of device characteristics and parameters, including the transfer, output, and diode characteristics, as well as parameters such as drain leakage current (*I*_dss_), breakdown voltage (*BV*_dss_), and on-resistance (*R*_DSon_), was observed. Switching performance was verified using a double-pulse test, where a significant change in switching times was observed. These changes during the lifetime operation of the device pose considerable risks against the requirement for the long-term reliability of modern power devices. Here we aim for the application of well-accepted UIS stress on SiC-based TrenchMOSFET with a detailed study of trap states localized within the energy gap. The mutual comparison of device electrical parameters and trap states in the band diagram illustrates the impact of UIS stress.

## 2. Experimental

Tested devices were commercially available 1.2-kV SiC TrenchMOSFET devices produced by Infineon (Dresden, Germany) with rated current 13 A and a typical value of on-resistance, *R*_DSon_ = 220 mΩ. The structure of the device is vertical, with an asymmetric channel oriented along a plane, which gives the best channel mobility and lowest interface trap density [7]. Two deep p-wells serve as an anode of the body diode for freewheeling operation, while one of the p-wells also shields the gate structure from high potential and decreases the risk of gate dielectric breakdown. Devices are encapsulated in the PG-TO247-4 package.

**Figure 1:**
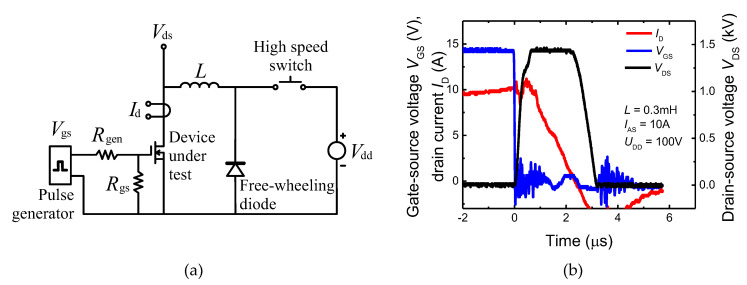
UIS test: (**a**) Basic UIS test circuit. (**b**) Typical current and voltage waveforms of SiC MOSFET under UIS test conditions.

**Figure 2:**
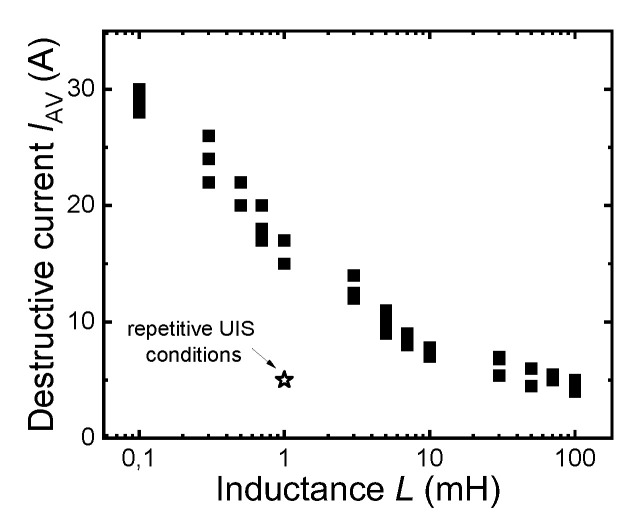
Measured destructive currents of investigated SiC TrenchMOS samples for different inductive loads. The star symbol represents the stress current induced during the repetitive UIS test.

Commercial UIS tester ITC55100 (Integrated Technology Corporation, Tempe, USA)was used for UIS testing. The entire testing procedure, as well as the layout of the test circuit, were defined by JEDEC standard—JESD24-5. Figure 1a,b show the circuit diagram and the typical voltage and current waveforms during the SiC MOSFET test. The device under test (DUT) is connected to the power supply through the inductor and the high-side switch (HSW). When HSW is shorted, and DUT is turned on, the current limited by the inductor starts to rise linearly. DUT and HSW are turned off when the current (*I*_D_) reaches the required (pre-set) value in the experiments. The magnetic field in the inductor (*L*) induces a counter-electromotive force that can build up surprisingly high potentials across the switch (DUT). If no protective circuits are added to the switch, all the energy accumulated in the inductor is dissipated directly in the device switch. To correctly set parameters for repetitive UIS tests, the first single pulse UIS capability of tested devices was verified. Obtained values of destructive currents for single pulse operation, measured for different inductances, are shown in Figure 2. Observed results are in good agreement with basic UIS theory, where if the intrinsic temperature of the blocking PN junction is assumed as a limit value for the passive mode of destruction and tests start at a constant temperature, then we can write the relation between the inductance, *L*, and the critical value of the avalanche current, *I*_Avcrit_, as [8]:

(1) ΔTM=23Cth0.IAV.VBReff.LIAVVBReff2.2 → IAVcrit ~ 1L3.2 

## 3. Repetitive UIS Stress

### 3.1. Impact on I-V and C-V Characteristics

Based on the results from single pulse UIS measurements, conditions for the repetitive UIS stress were set with a significantly lower avalanche current than the critical value for the given inductance. Parameters were set as follows: load inductance (*L*) = 1 mH, supply voltage (*V*_DD_) = 100 V, switched current (*I*_AS_) = 5 A. These parameters have been determined in accordance with the recommendation given by the application note AN-1005 [9]. The switching period between two pulses was set to 5 ms, and an additional time of 800 ms was set after every 100 sets of pulses. The current and switching period was set according to the maximum non-destructive single pulse current for a given inductive load and to minimize heat accumulation in samples. However, during switching, significant heat is still generated in transistors; therefore, all I-V measurements were performed after the device cooled down for ten minutes to prevent the impact of temperature on device parameters shifting.

**Figure 3:**
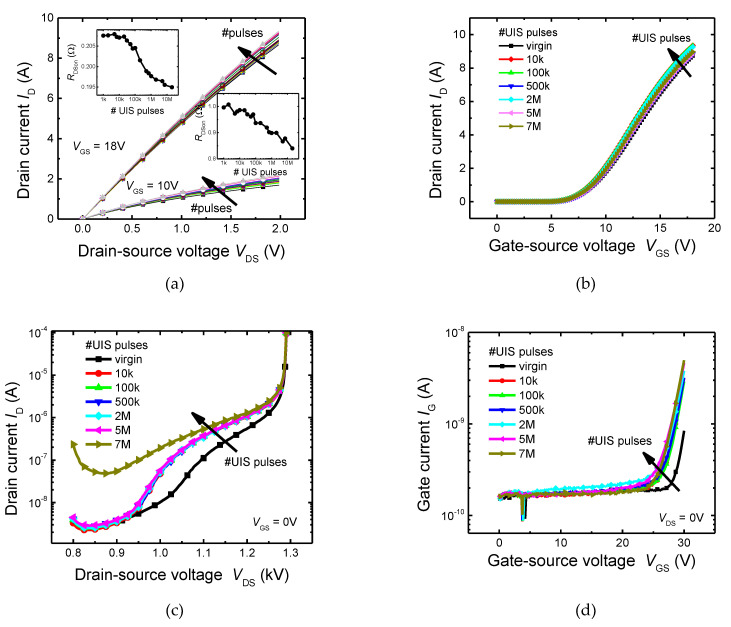

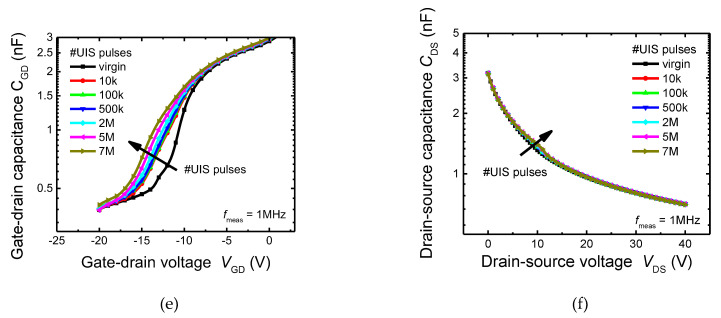
Measured the impact of repetitive UIS on the stability of electrical characteristics. (**a**) Output characteristics and on-resistance (inset graph), various colours of output characteristics stand for different numbers of UIS pulses, (**b**) transfer characteristics, (**c**) drain leakage current and breakdown, (**d**) gate leakage current, (**e**) gate–drain capacitance, (**f**) drain–source capacitance.

First, we analyzed the impact of repetitive UIS on static I-V characteristics. Device characteristics were measured before and after each sequence of pulses to observe the gradual effects of repetitive UIS stress. Measured I-V characteristics for virgin and stressed samples, up to 7 million pulses, were shown in Figure 3. Observations were as follows: Repetitive UIS pulses induced shifts in all analyzed I-V characteristics. The output and transfer characteristics’ shifts were mostly uniform (Figure 3a,b). We observed a slight negative shift of the threshold voltage. This negative shift of the threshold voltage is obviously also responsible for the decline of the on-resistance, evident in the output characteristics. The on-resistance has decreased from the initial value of 207 mΩ, before the stress, to the value of 195 mΩ after 7 million pulses. The decrease in on-resistance clearly points to the fact that the characteristics are not affected by thermal cycling, but other mechanisms have to be present. It is a fact that during the avalanching phase of the UIS pulse, large concentrations of high energy carriers are generated close to blocking the PN junction. Therefore, a negative shift of threshold voltage indicates the trapping of a positive charge on the channel-gate interface or in its vicinity. The origin of this positive charge can be attributed to the high energy holes generated during the UIS pulse. Our assumption is confirmed by a significant change in the gate leakage current (Figure 3d), which must be caused by the formation or activation of electrically active defects in the gate dielectric. A significant change in the concentration of electrically active defects in the vicinity of the blocking PN junction is also indicated by a significant change in the drain-to-source leakage current (Figure 3c). However, no change in static breakdown voltage value was observed. The change in the leakage current characteristics (Figure 3c), which is caused by an increase in the concentration of electrically active traps, is manifested by two things, an increase in the leakage current itself and a change in the shape of the I-V curve, which is most likely related to a change in the distribution of the electric field due to a change in the charge distribution.

Another important observation was the shift of static C-V characteristics (Figure 3e,f). While only minimal change was observed in the drain-source capacitance characteristic, *C*_DS_, which is primarily determined by the capacity of the blocking PN junction, a significant change in the gate-to-drain capacitance, *C*_GD_, was observed. It is caused by the fact that the bottom part of the trench gate electrode, which determines the *C*_GD_ capacity, is strongly exposed to hot carriers generated during UIS stress.

### 3.2. Impact on Switching Performance

**Figure 4:**
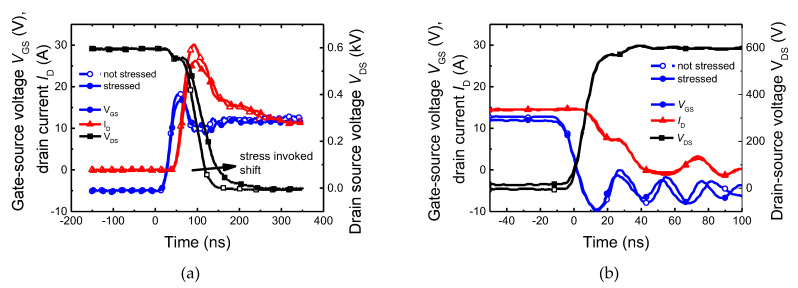
Impact of repetitive UIS on switching performance of the power SiC TrenchMOS. (**a**) Turn-on and (**b**) turn-off the process. A significant increase in turn-on time was observed after 10 million UIS pulses. No impact on the turn-off process was observed. Open symbols mean the values correspond to virgin samples, while the filled symbols represent the measurement after 10 million UIS pulses.

Next, switching processes (turn-on and -off) were analyzed using a double-pulse tester. Waveforms of the gate and drain voltages and drain current are shown in Figure 4. First, measurements were performed on virgin samples. Next, samples were exposed to 10 million UIS pulses. After repetitive UIS stress, a significant increase in switching time for the turn-on process was observed. However, no impact on switching time was observed for the turn-off process. The explanation may be that defects are generated close to or on the gate-drain interfaces. These defects are charged when the transistor is in an off state and high voltage is present between the gate and drain. Then, when the transistor is turned on, discharging of these defects slows down the drain voltage decrease. A similar change in the dynamic behaviour was previously observed and described by T. Laska et al. in [10] for IGBTs, where authors assumed that carriers accelerated by high dynamic electrical fields were injected into the gate oxide. As a result, charges at or near the interface Si/oxide over the whole trench shape below the p-body region are formed, leading to an increase in the turn-off delay time. The charge trapping takes an extremely short time (in the ns region), while the discharging process is drastically slower (in the µs and ms region). As a result, both the turn-on and turn-off processes are affected by the charge trap states; however, the observation of the switching process in the ns region can reveal the charge trapping only, while the charge detrapping is out of the observed time range. The charge detrapping (charge release) was analyzed in detail by the deep-level transient spectroscopy that is suitable for long-living states only.

## 4. Defect Analysis

**Figure 5:**
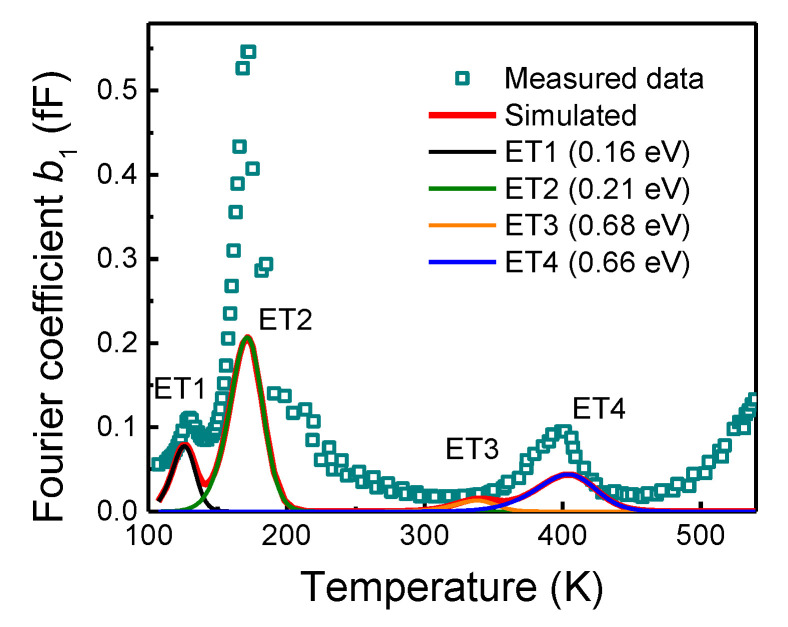
Fourier coefficient, *b*_1_, spectrum before the stress. The position of evaluated deep levels is verified by the simulation (solid lines).

The structural defects originated in the device fabrication process or due to device stress, serve as charge carrier traps. The charge trap identification was performed using the deep-level transient Fourier spectroscopy (DLTFS) system DL8000 (BIO-RAD Micromeasurement, UK). The gate-drain capacitance transients, *C*_GD_, were recorded since the channel region stands for the most affected capacitance contribution by repetitive UIS stress and different sets of parameters, and that is all. Note that the DLTFS method relies on the observation of a transient response on an excitation pulse applied at various temperatures. As a result, the temperature dependence of transients’ relaxation time reveals the activation energies of hidden charge traps. The measurement conditions, such as excitation pulse amplitude or time, were conserved for all measurements to achieve fully comparable parameters for the virgin device and device after the stress. The Fourier transform analysis was employed to analyze the multiexponential transients. Fourier coefficients, *a*_1_ and *b*_1_, representing the cosine and sine coefficients of the first order, were estimated for the Arrhenius plots evaluation [11]. First, the virgin device was analyzed to map the traps’ energy distribution before the stress application, as shown in Figure 5. Interestingly, four dominant electron trap states denoted as ET1 to ET4 were observed. The Arrhenius plot was applied to extract the traps’ parameters, such as the activation energy and the capture cross-section, as shown in Table 1. A numerical simulation was used to verify the evaluated parameters of each trap state. All the measurements were summarized in the Supplementary Material, Figure S1.

Note that even though the activation energy derived from transient spectroscopy is not an actual position of the energy level within the energy band gap due to the temperature-dependent cross-section, many reports found relations between the activation energies and structural defects causing the trap states. For example, metal impurities are a common source of electron traps originating from the imperfection of the MOSFET fabrication technology. The electron traps, ET1 and ET2, are associated with the presence of Cr, Ti, or W, since these impurities create localized states, 0.13 to 0.18 eV from the bottom edge of the conduction band, *E*_c_ [12,13,14,15]. Electron trap, ET3, is a known intrinsic defect denoted as $Z_{1,2}$ and stands for the negative U-center, the defect that can exist in more than a single charge state. Even though it was assigned to the carbon vacancy as the most common structural defect in 4H-SiC, Hemmingsson et al. [15] had shown that it consists of two contributions corresponding to the emission of two electrons from the $Z_{1}^{0/+}$ and $Z_{2}^{0/+}$ levels [12,13,16,17,18,19,20]. These defects were discussed as (=/0) transitions of carbon vacancy, $V_{C}^{}$, located at the hexagonal (*−h*) and pseudo-cubic (*−k*) sites [12,13]. However, under common conditions, we observe only the $Z_{1,2}^{–/+}$ transition, while the $Z_{1}^{–/0}$ and $Z_{2}^{–/0}$ levels with activation energies of about 0.52 and 0.45 eV, respectively, are not observable [15]. As a result, the $Z_{1,2}^{–/+}$ transition is an origin of a strongly asymmetric peak in the spectrum that can be deconvoluted into two energy levels close to each other. Interestingly, the Laplace DLTS was used to separate the $Z_{1,2}^{–/+}$ transition into two emissions with different emission times since it represents a two-stage process ($V_{C}^{=}\to V_{C}^{–}+e\to V_{C}^{0}+2e$), and conventional DLTS merges them into a single process. Here, the first stage, $Z_{1}$, reached an energy level of 0.58 eV, while the second stage, $Z_{2}$, was estimated at 0.65 eV. In other words, these two negative U-centres have one donor and one acceptor level. The donor levels, $Z_{1}^{0}$ and $Z_{2}^{0}$, have activation energies of 0.58 and 0.45 eV, respectively, whereas the acceptor levels, $Z_{1}^{–}$ and $Z_{2}^{–}$, exhibit activation energies of 0.67 and 0.71 eV, respectively [16]. Therefore, we assume that the electron traps, ET3 and ET4, are related to the $Z_{1,2}$ transition and stand for $Z_{1}^{–}$ and $Z_{2}^{–}$ transitions.

Next, the device was exposed to two different stress conditions: short repetitive UIS stress with a duration of 1 h and long repetitive UIS stress with a duration of 24 h. Finally, to distinguish stress impact, another device for trap state analysis was also prepared and exposed to static stress for 24 h. During this static stress, the device was kept in the drain-to-source breakdown with a constant drain current of 100 µA.

**Figure 6:**
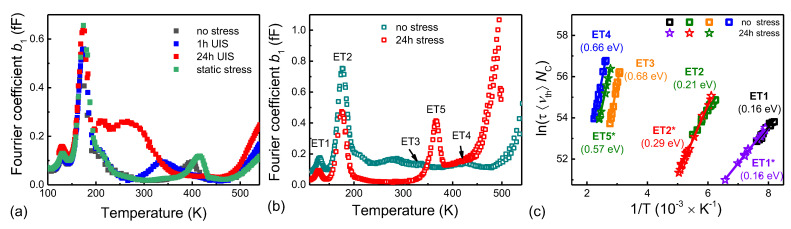
(**a**) Comparison of the Fourier coefficient *b*_1_ spectra induced by different stress conditions and (**b**) for the virgin device and after repetitive UIS stress with a duration of 24 h. (**c**) Arrhenius plot of evaluated trap states.

The Fourier coefficient *b*_1_’s spectra and Arrhenius plot, shown in Figure 6a, illustrated the static stress effect. As a result, we can summarize these observations: As the spectra for the unstressed virgin device and the device after static stress are almost identical, we can conclude that static stress had minimal impact on the device degradation. On the other hand, both repetitive UIS stress considerably affected the observed spectra. The concentration of the trap ET1 was slightly increasing with the length of UIS stress, while the increase in the trap state ET2 was observed only for the long-term stress (24 h). The highest impact of repetitive UIS was observed on trap states ET3 and ET4. First, after the short stress (1 h), the signal from trap state ET3 was increased. However, the long UIS stress had the opposite effect, and the signal from the trap ET3 was significantly suppressed. Similarly, the signal from trap ET4 is suppressed after the long-term UIS stress. Using different measurement conditions, we identified one newly generated electron trap state, ET5, with an activation energy of 0.57 eV. Since the emission from trap state ET5 is present without signals from ET3 and ET4, we can conclude that it is the first stage of the two-electron $Z_{1,2}\;$ process ($Z_{1}^{0}\;$ transition). Furthermore, we assume that the trap state was activated by the stress conditions because it was observable only after long-term UIS stress.

## 5. Conclusions

The gradual degradation of commercial trench gate 1.2 kV 4H-SiC MOSFETs produced by Infineon, subjected to the repetitive avalanche pulses, was measured and analyzed. The ITC55100 UIS tester was utilized to generate the stress. Devices were exposed to up to 7 million stress pulses and stressed for up to 24 h. The strong impact of repetitive avalanching stress on the electrical performance of tested devices was observed. After repetitive UIS stress tests, devices showed a consistent degradation tendency characterized by a decreased threshold voltage (*V*_th_), decreased on-resistance (*R*_DSon_), and a significant increase in drain-source and gate-source leakage currents. Moreover, a significant shift of gate-to-drain capacitance (*C*_DG_) was observed. Long-term repetitive UIS stress also invoked a significant increase in turn-on time. The trapping was also analyzed using transient capacitance spectroscopy. Four electron traps were evaluated from the measured spectra of the virgin device. Numerical simulations were applied to verify their parameters, and the trap states were associated with metal impurities or charge transitions of carbon vacancies. A significant shift of Fourier coefficients *b*_1_ spectra and new electron trap state ET5 was observed after long-term repetitive UIS stress. The newly generated state was assumed to be a first-stage charging of the two-stage $Z_{1,2}\;$ transition related to the multiple charge states of carbon vacancy. The analysis confirmed the significant impact of repetitive avalanche stress on the overall stability of electrical parameters of the power SiC MOSFET and pointed to a possible negative impact on its overall long-term reliability due to the impact on traps capable of multiple charge states. The results point out that the device resistance to UIS stress can be improved by fabrication technology improvements that will suppress the presence of interface states and structural defects.

## Figures and Tables

**Table 1:** Parameters of evaluated trap states. The asterisk (*) denotes the trap after stress conditions. The capture cross-section has an evaluated range of one standard deviation.

Trap	*E*_c_-*E*_t_ (eV)	σ (cm^2^)	Possible Origin
ET1	0.16 ± 0.01	1.27 × 10^−17^ (0.39~3.91 × 10^−17^)	metal impurity
ET1*	0.16 ± 0.00	1.11 × 10^−17^ (0.81~1.52 × 10^−17^)	
ET2	0.21 ± 0.01	4.06 × 10^−18^ (2.44~6.75 × 10^−18^)	metal impurity
ET2*	0.29 ± 0.00	1.12 × 10^−15^ (0.88~1.44 × 10^−15^)	
ET3	0.68 ± 0.01	1.37 × 10^−14^ (0.86~2.18 × 10^−14^)	$Z_{1,2}\;$ transition ($Z_{1}^{–}$)
ET4	0.66 ± 0.01	1.11 × 10^−16^ (0.73~1.69 × 10^−16^)	$Z_{1,2}\;$ transition ($Z_{2}^{–}$)
ET5*	0.57 ± 0.02	3.39 × 10^−17^ (1.92~5.99 × 10^−17^)	$Z_{1,2}\;$ transition ($Z_{1}^{0}$)

## Data Availability

The data presented in this study are available in the Supplementary Materials.

## Supplementary Materials

The following supporting information can be downloaded at: https://www.mdpi.com/article/10.3390/ma15228230/s1, Figure S1: Deep-level transient spectroscopy data.
