# Supplementary material for: Charge Trap States of SiC Power TrenchMOS Transistor under Repetitive Unclamped Inductive Switching Stress
Source: Materials (Basel). 2022 Nov 19;15(22):8230. doi: 10.3390/ma15228230 (PMC9697379; doi:10.3390/ma15228230)
Supplement: Supplementary file 1 [file materials-15-08230-s001.zip › materials-2005591-supplementary.pdf]

materials

Supplementary material

Charge Trap States of SiC Power TrenchMOS Transistor under Repetitive UIS Stress

Juraj Marek [1,*], Jozef Kozarik [1,2], Michal Minarik [1], Ales Chvala [1], Matej Matus [1], Martin Donoval [1], Lubica Stuchlikova [1] and Martin Weis [1]

[1] Institute of Electronics and Photonics, Slovak University of Technology in Bratislava, Ilkovičova 3, 812 19 Bratislava, Slovakia
[2] NanoDesign, s.r.o, Drotárska 19a, 811 04 Bratislava, Slovakia
* Correspondence: juraj.marek@stuba.sk

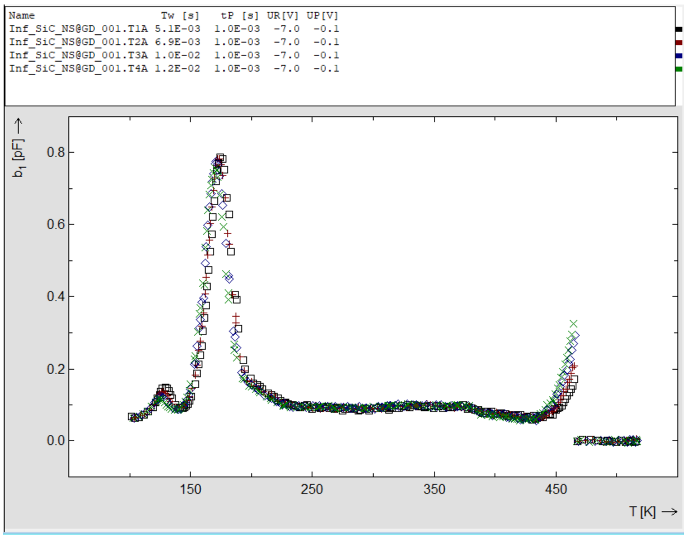

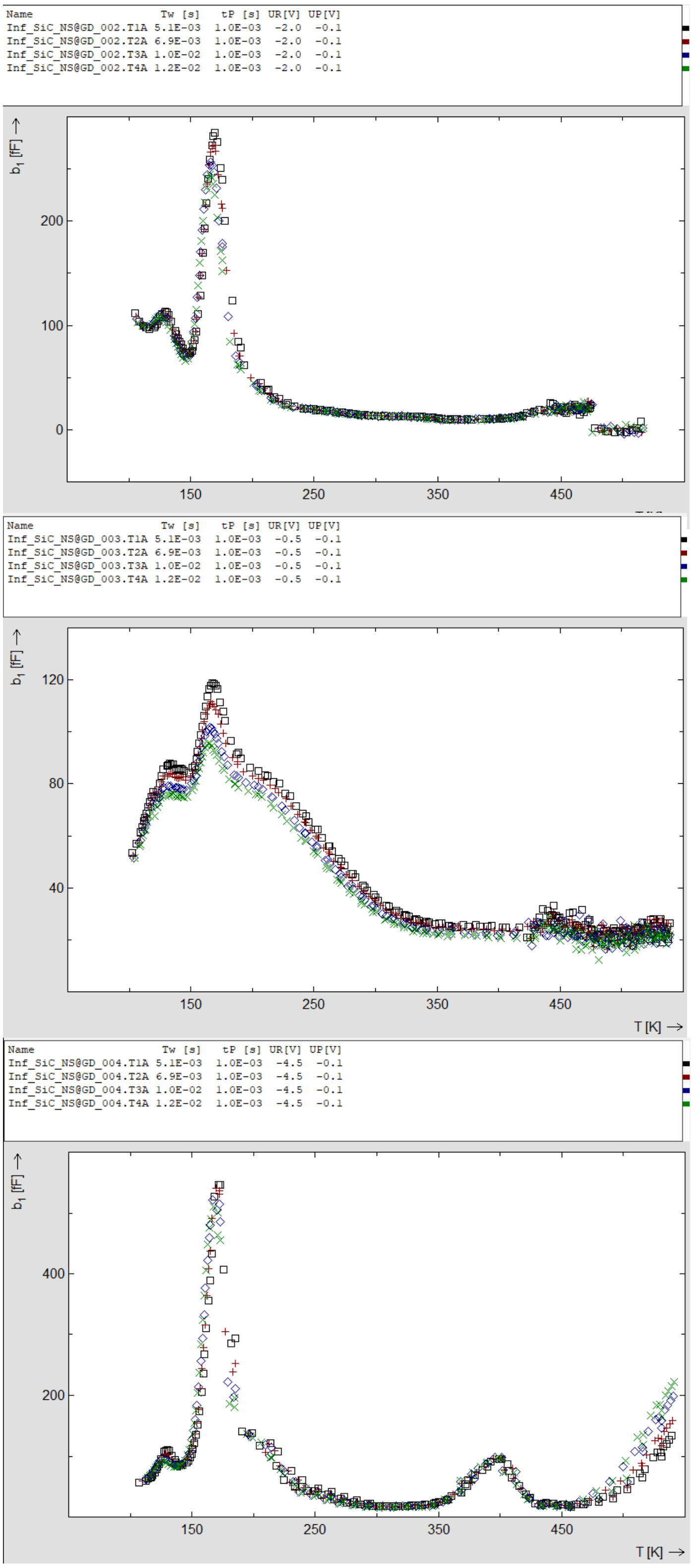
Name Tw [s] tP [s] UR[V] UP[V]
Inf_SiC_NS@GD_002.T1A 5.1E-03 1.0E-03 -2.0 -0.1
Inf_SiC_NS@GD_002.T2A 6.9E-03 1.0E-03 -2.0 -0.1
Inf_SiC_NS@GD_002.T3A 1.0E-02 1.0E-03 -2.0 -0.1
Inf_SiC_NS@GD_002.T4A 1.2E-02 1.0E-03 -2.0 -0.1
b1 [fF] →
200
100
0
150
250
350
450
Name Tw [s] tP [s] UR[V] UP[V]
Inf_SiC_NS@GD_003.T1A 5.1E-03 1.0E-03 -0.5 -0.1
Inf_SiC_NS@GD_003.T2A 6.9E-03 1.0E-03 -0.5 -0.1
Inf_SiC_NS@GD_003.T3A 1.0E-02 1.0E-03 -0.5 -0.1
Inf_SiC_NS@GD_003.T4A 1.2E-02 1.0E-03 -0.5 -0.1
b1 [fF] →
120
80
40
150
250
350
450
T [K] →
Name Tw [s] tP [s] UR[V] UP[V]
Inf_SiC_NS@GD_004.T1A 5.1E-03 1.0E-03 -4.5 -0.1
Inf_SiC_NS@GD_004.T2A 6.9E-03 1.0E-03 -4.5 -0.1
Inf_SiC_NS@GD_004.T3A 1.0E-02 1.0E-03 -4.5 -0.1
Inf_SiC_NS@GD_004.T4A 1.2E-02 1.0E-03 -4.5 -0.1
b1 [fF] →
400
200
150
250
350
450
T [K] →

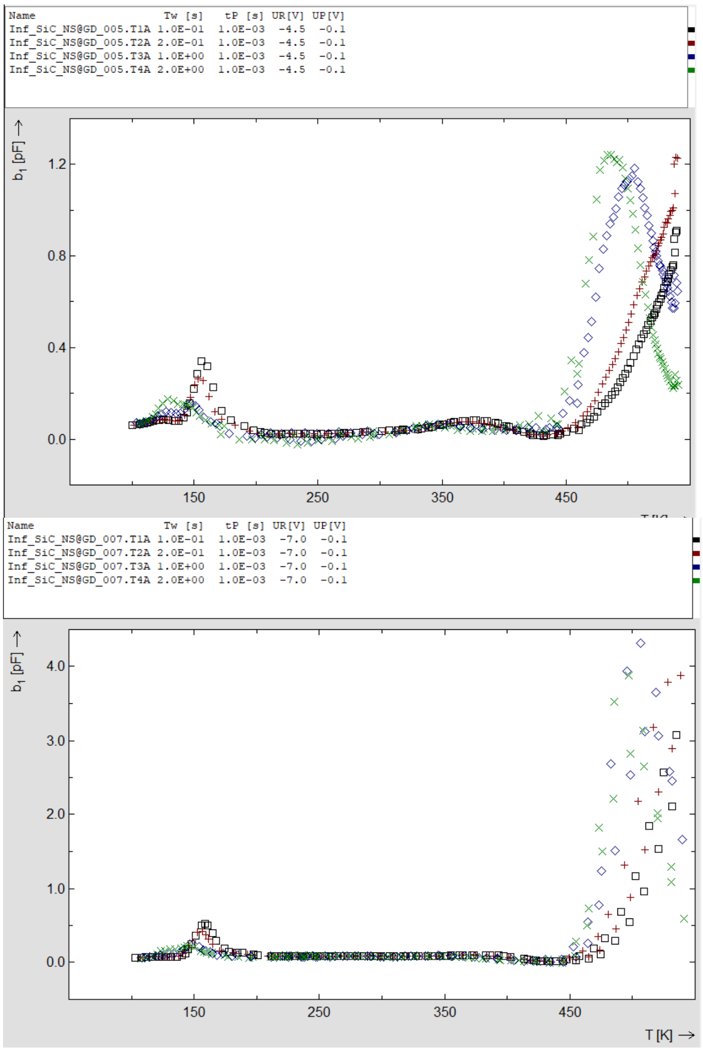
Name Tw [s] tP [s] UR[V] UP[V]
Inf_SiC_NS@GD_005.T1A 1.0E-01 1.0E-03 -4.5 -0.1
Inf_SiC_NS@GD_005.T2A 2.0E-01 1.0E-03 -4.5 -0.1
Inf_SiC_NS@GD_005.T3A 1.0E+00 1.0E-03 -4.5 -0.1
Inf_SiC_NS@GD_005.T4A 2.0E+00 1.0E-03 -4.5 -0.1
b_1 [pF] →
1.2
0.8
0.4
0.0
150
250
350
450
Name Tw [s] tP [s] UR[V] UP[V]
Inf_SiC_NS@GD_007.T1A 1.0E-01 1.0E-03 -7.0 -0.1
Inf_SiC_NS@GD_007.T2A 2.0E-01 1.0E-03 -7.0 -0.1
Inf_SiC_NS@GD_007.T3A 1.0E+00 1.0E-03 -7.0 -0.1
Inf_SiC_NS@GD_007.T4A 2.0E+00 1.0E-03 -7.0 -0.1
b_1 [pF] →
4.0
3.0
2.0
1.0
0.0
150
250
350
450
T [K] →

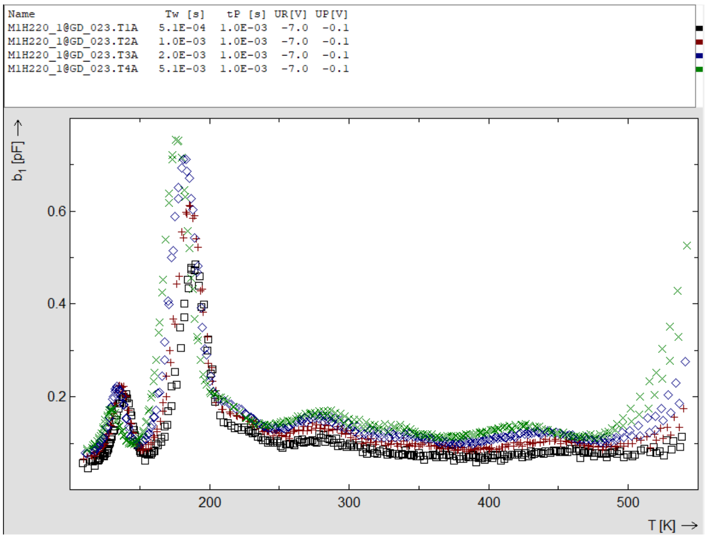
Name Tw [s] tP [s] UR[V] UP[V]
M1H220_1@GD_023.T1A 5.1E-04 1.0E-03 -7.0 -0.1
M1H220_1@GD_023.T2A 1.0E-03 1.0E-03 -7.0 -0.1
M1H220_1@GD_023.T3A 2.0E-03 1.0E-03 -7.0 -0.1
M1H220_1@GD_023.T4A 5.1E-03 1.0E-03 -7.0 -0.1
b_1 [pF] →
0.6
0.4
0.2
200
300
400
500
T [K] →

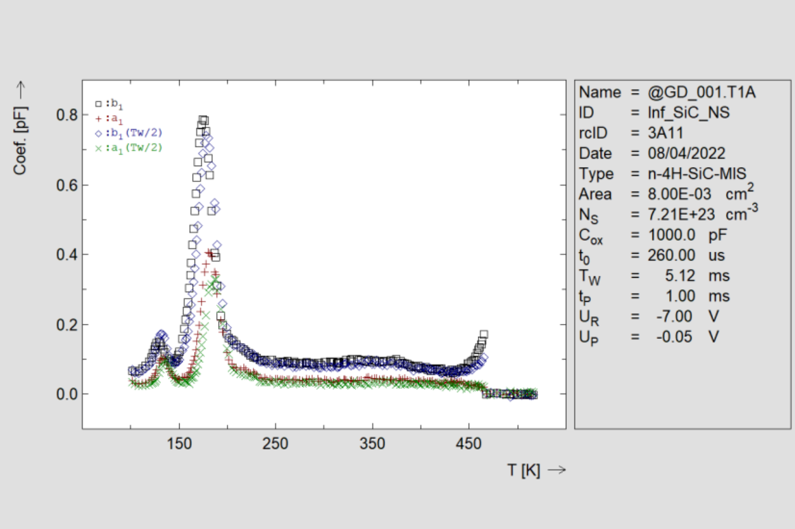
Coef. [pF] →
0.8
0.6
0.4
0.2
0.0
□ :b1
+ :a1
◇ :b1 (Tw/2)
× :a1 (Tw/2)
150
250
350
450
T [K] →
Name = @GD_001.T1A
ID = Inf_SiC_NS
rcID = 3A11
Date = 08/04/2022
Type = n-4H-SiC-MIS
Area = 8.00E-03 cm2
NS = 7.21E+23 cm-3
Cox = 1000.0 pF
t0 = 260.00 us
TW = 5.12 ms
tP = 1.00 ms
UR = -7.00 V
UP = -0.05 V

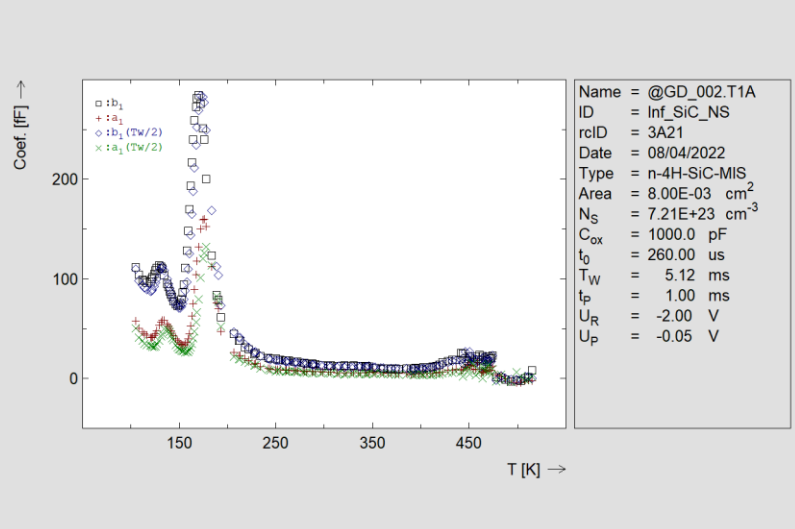
Coef. [fF] →
200
100
0
□ :b1
+ :a1
◇ :b1 (Tw/2)
× :a1 (Tw/2)
150
250
350
450
T [K] →
Name = @GD_002.T1A
ID = Inf_SiC_NS
rcID = 3A21
Date = 08/04/2022
Type = n-4H-SiC-MIS
Area = 8.00E-03 cm2
NS = 7.21E+23 cm-3
Cox = 1000.0 pF
t0 = 260.00 us
TW = 5.12 ms
tP = 1.00 ms
UR = -2.00 V
UP = -0.05 V

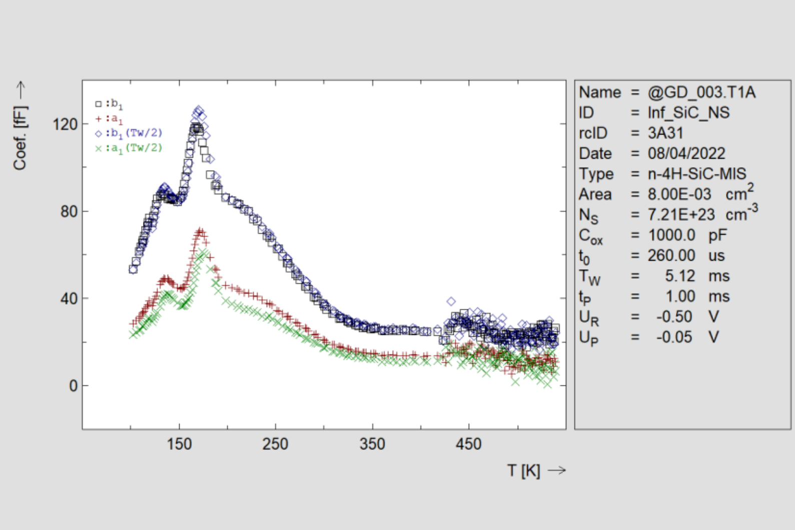
Coef. [fF] →
120
80
40
0
□ :b1
+ :a1
◇ :b1 (Tw/2)
× :a1 (Tw/2)
150
250
350
450
T [K] →
Name = @GD_003.T1A
ID = Inf_SiC_NS
rcID = 3A31
Date = 08/04/2022
Type = n-4H-SiC-MIS
Area = 8.00E-03 cm2
NS = 7.21E+23 cm-3
Cox = 1000.0 pF
t0 = 260.00 us
TW = 5.12 ms
tP = 1.00 ms
UR = -0.50 V
UP = -0.05 V

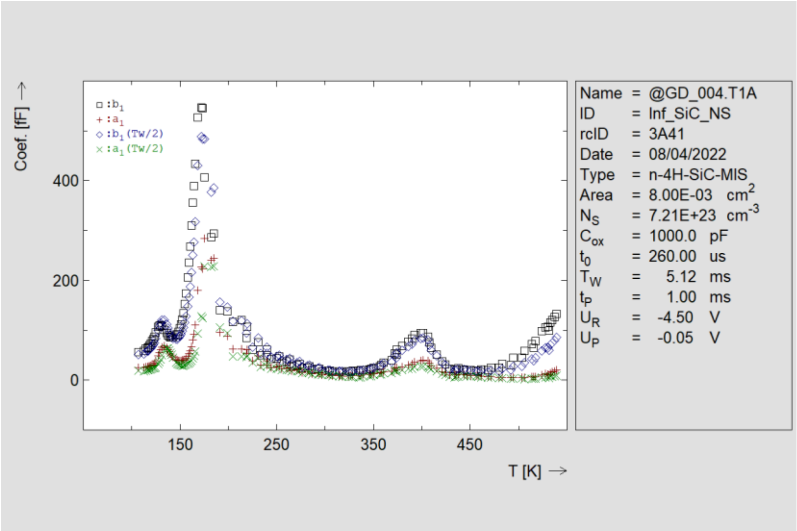
□ :b1
+ :a1
◇ :b1 (Tw/2)
× :a1 (Tw/2)
Coef. [fF] →
400
200
0
150
250
350
450
T [K] →
Name = @GD_004.T1A
ID = Inf_SiC_NS
rcID = 3A41
Date = 08/04/2022
Type = n-4H-SiC-MIS
Area = 8.00E-03 cm2
NS = 7.21E+23 cm-3
Cox = 1000.0 pF
t0 = 260.00 us
TW = 5.12 ms
tP = 1.00 ms
UR = -4.50 V
UP = -0.05 V

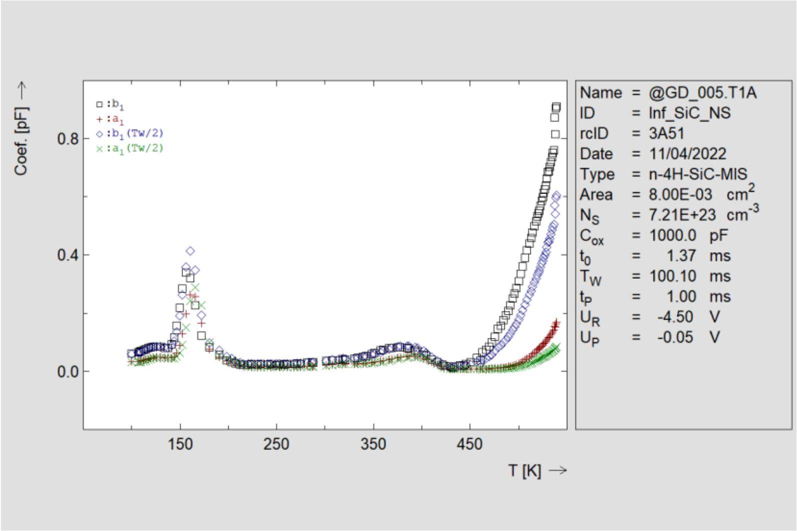
□ :b1
+ :a1
◇ :b1 (Tw/2)
× :a1 (Tw/2)
Coef. [pF] →
0.8
0.4
0.0
150
250
350
450
T [K] →
Name = @GD_005.T1A
ID = Inf_SiC_NS
rcID = 3A51
Date = 11/04/2022
Type = n-4H-SiC-MIS
Area = 8.00E-03 cm2
NS = 7.21E+23 cm-3
Cox = 1000.0 pF
t0 = 1.37 ms
TW = 100.10 ms
tP = 1.00 ms
UR = -4.50 V
UP = -0.05 V

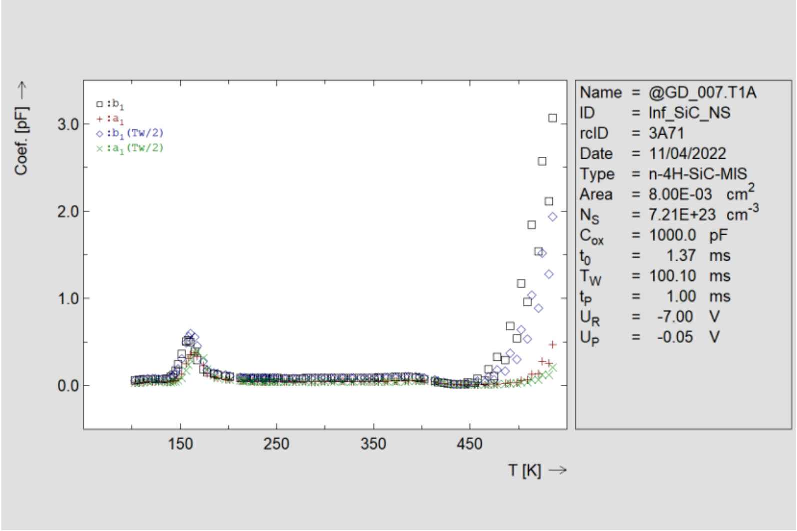
□ :b1
+ :a1
◇ :b1 (Tw/2)
× :a1 (Tw/2)
Coef. [pF] →
3.0
2.0
1.0
0.0
150
250
350
450
T [K] →
Name = @GD_007.T1A
ID = Inf_SiC_NS
rcID = 3A71
Date = 11/04/2022
Type = n-4H-SiC-MIS
Area = 8.00E-03 cm2
NS = 7.21E+23 cm-3
Cox = 1000.0 pF
t0 = 1.37 ms
TW = 100.10 ms
tP = 1.00 ms
UR = -7.00 V
UP = -0.05 V

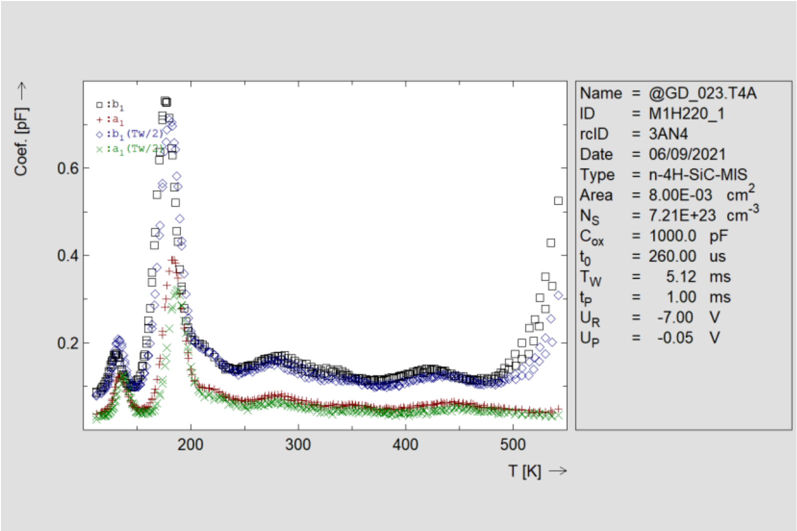

Measurements on virgin samples

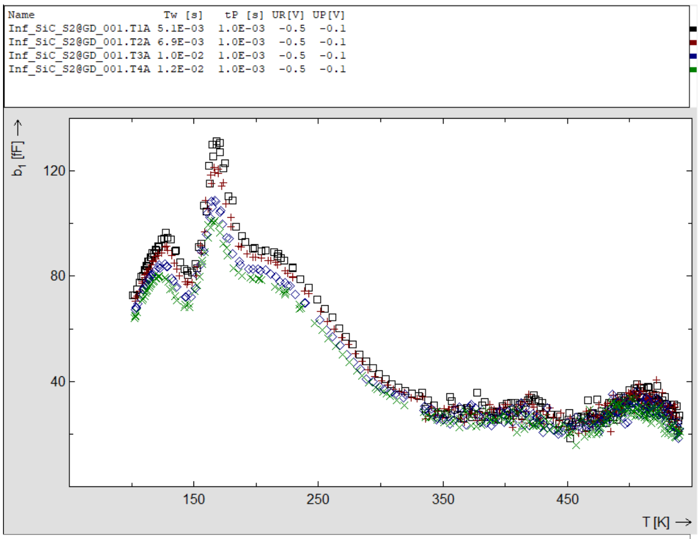

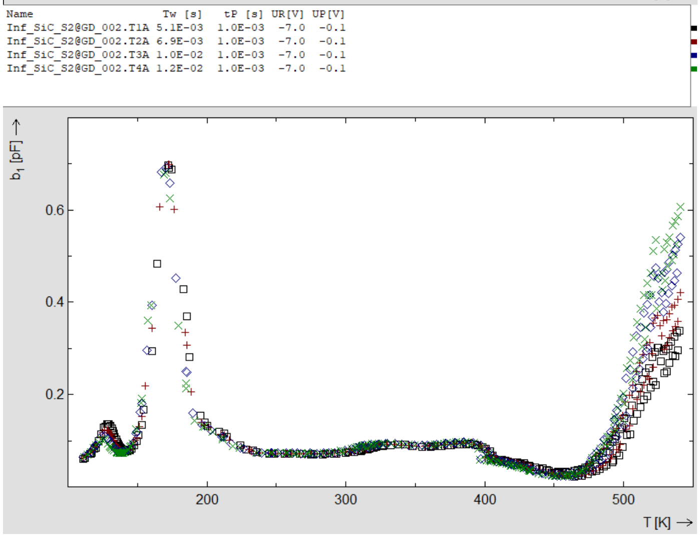

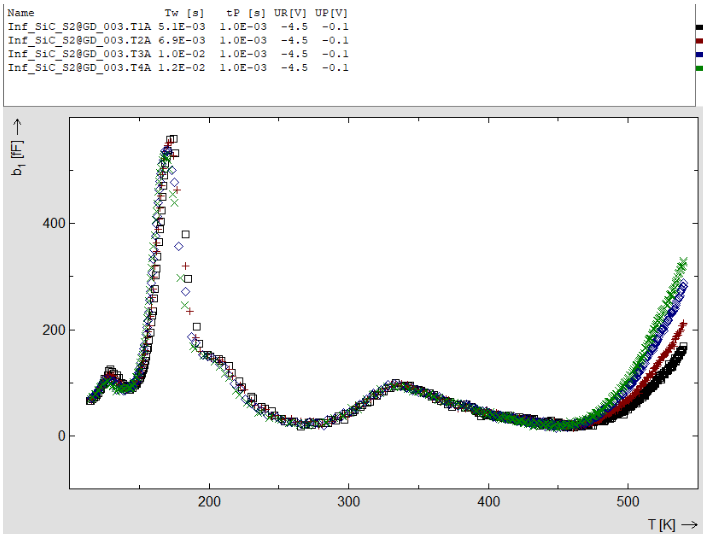

Name Tw [s] tP [s] UR[V] UP[V]
Inf_SiC_S2@GD_003.T1A 5.1E-03 1.0E-03 -4.5 -0.1
Inf_SiC_S2@GD_003.T2A 6.9E-03 1.0E-03 -4.5 -0.1
Inf_SiC_S2@GD_003.T3A 1.0E-02 1.0E-03 -4.5 -0.1
Inf_SiC_S2@GD_003.T4A 1.2E-02 1.0E-03 -4.5 -0.1
b1 [fF] →
0
200
400
200
300
400
500
T [K] →

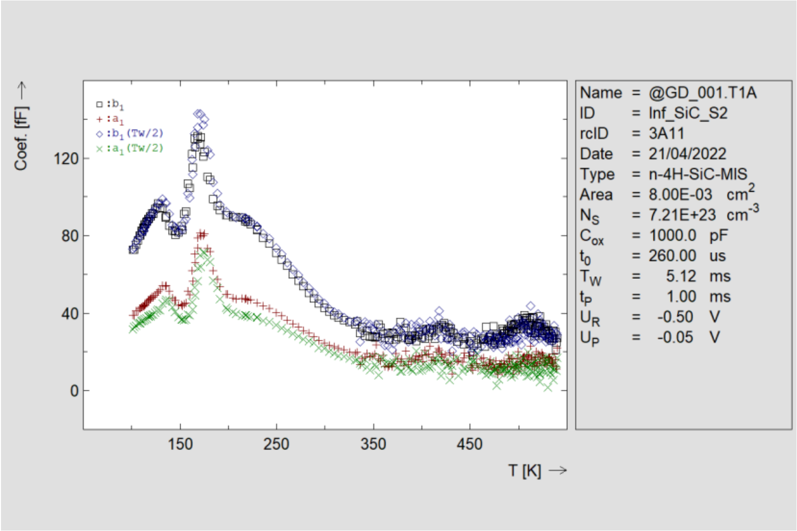

□ :b1
+ :a1
◇ :b1 (Tw/2)
× :a1 (Tw/2)
Coef. [fF] →
0
40
80
120
150
250
350
450
T [K] →
Name = @GD_001.T1A
ID = Inf_SiC_S2
rcID = 3A11
Date = 21/04/2022
Type = n-4H-SiC-MIS
Area = 8.00E-03 cm2
NS = 7.21E+23 cm-3
Cox = 1000.0 pF
t0 = 260.00 us
TW = 5.12 ms
tP = 1.00 ms
UR = -0.50 V
UP = -0.05 V

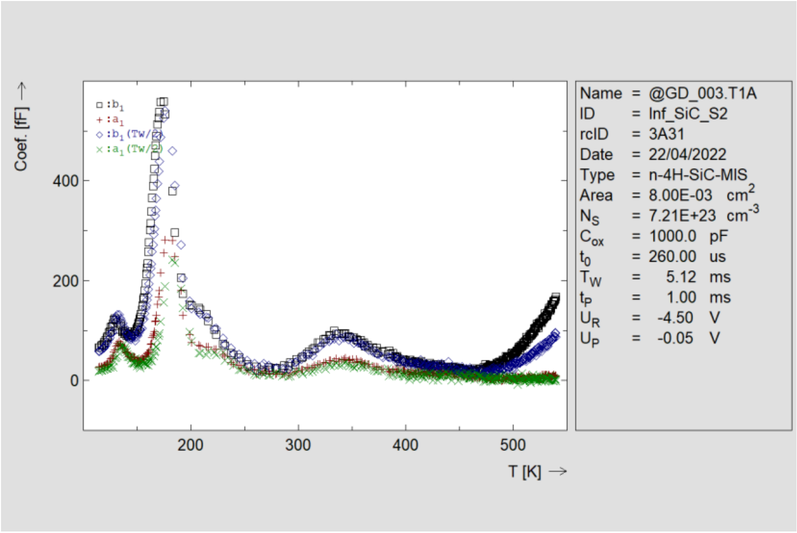

□ :b1
+ :a1
Coef. [fF] →
0
200
400
200
300
400
500
T [K] →
Name = @GD_003.T1A
ID = Inf_SiC_S2
rcID = 3A31
Date = 22/04/2022
Type = n-4H-SiC-MIS
Area = 8.00E-03 cm2
NS = 7.21E+23 cm-3
Cox = 1000.0 pF
t0 = 260.00 us
TW = 5.12 ms
tP = 1.00 ms
UR = -4.50 V
UP = -0.05 V

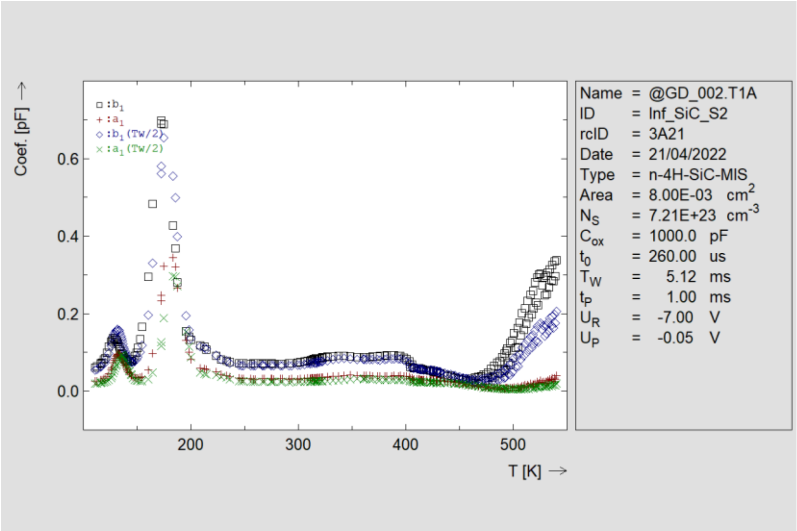

Measurements after 1 h of UISstress

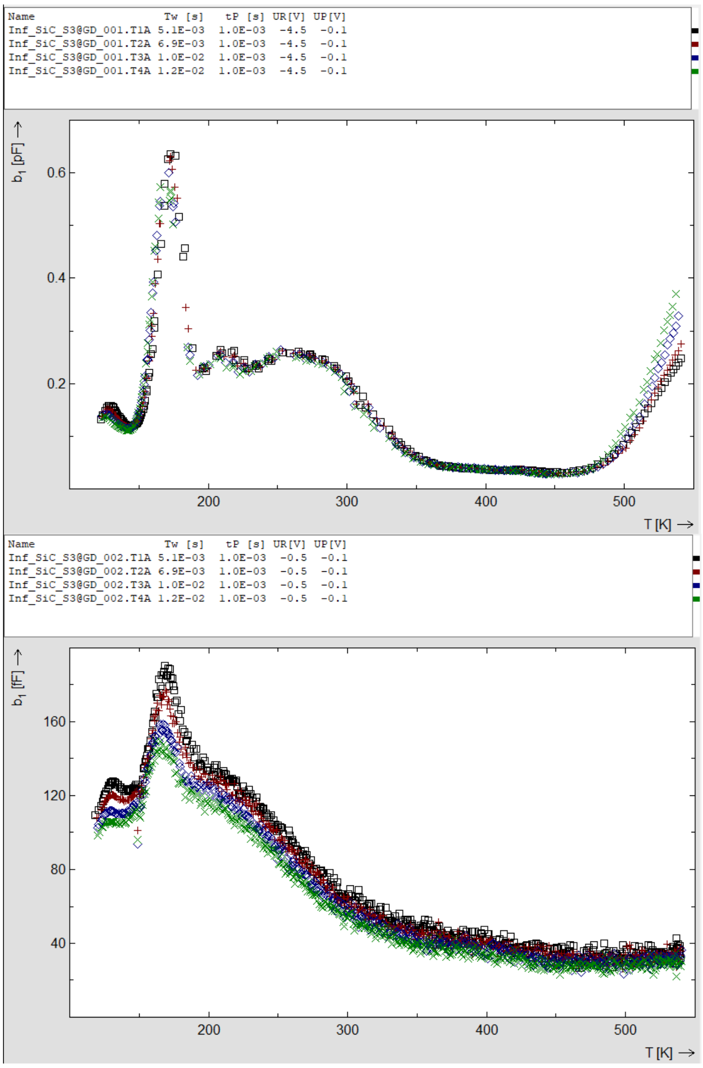

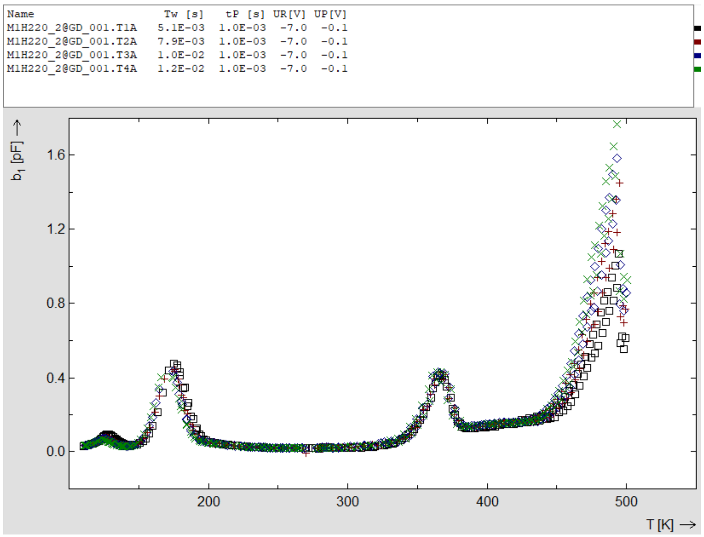
Name Tw [s] tP [s] UR[V] UP[V]
M1H220_2@GD_001.T1A 5.1E-03 1.0E-03 -7.0 -0.1
M1H220_2@GD_001.T2A 7.9E-03 1.0E-03 -7.0 -0.1
M1H220_2@GD_001.T3A 1.0E-02 1.0E-03 -7.0 -0.1
M1H220_2@GD_001.T4A 1.2E-02 1.0E-03 -7.0 -0.1
b1 [pF] →
1.6
1.2
0.8
0.4
0.0
200
300
400
500
T [K] →

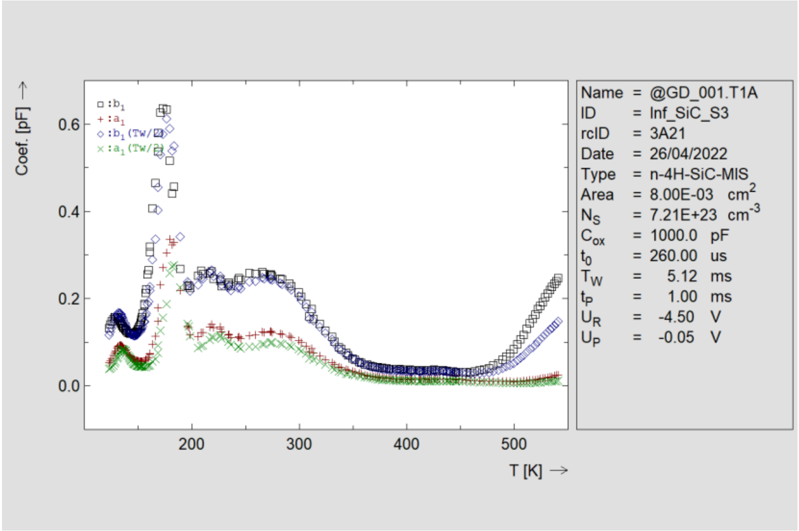
Coef. [pF] →
□ :b1
+ :a1
◇ :b1 (Tw/2)
× :a1 (Tw/2)
0.6
0.4
0.2
0.0
200
300
400
500
T [K] →
Name = @GD_001.T1A
ID = Inf_SiC_S3
rcID = 3A21
Date = 26/04/2022
Type = n-4H-SiC-MIS
Area = 8.00E-03 cm2
NS = 7.21E+23 cm-3
Cox = 1000.0 pF
t0 = 260.00 us
TW = 5.12 ms
tP = 1.00 ms
UR = -4.50 V
UP = -0.05 V

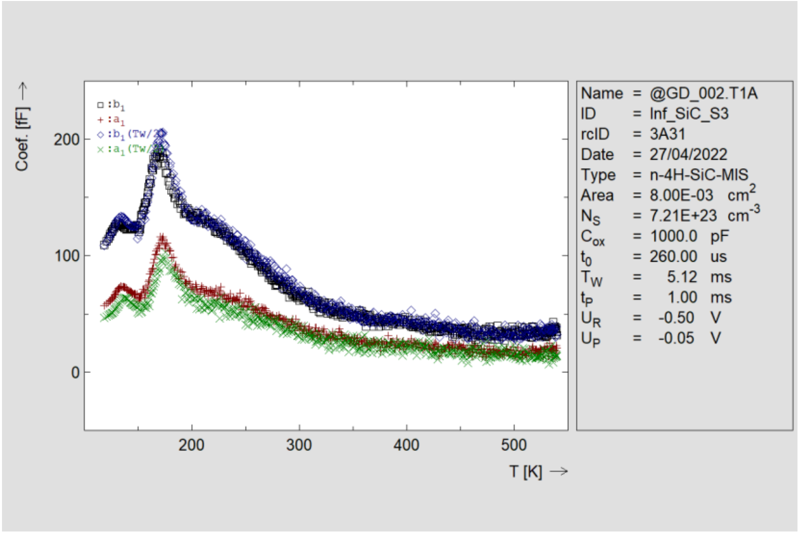
Coef. [fF] →
□ :b1
+ :a1
200
100
0
200
300
400
500
T [K] →
Name = @GD_002.T1A
ID = Inf_SiC_S3
rcID = 3A31
Date = 27/04/2022
Type = n-4H-SiC-MIS
Area = 8.00E-03 cm2
NS = 7.21E+23 cm-3
Cox = 1000.0 pF
t0 = 260.00 us
TW = 5.12 ms
tP = 1.00 ms
UR = -0.50 V
UP = -0.05 V

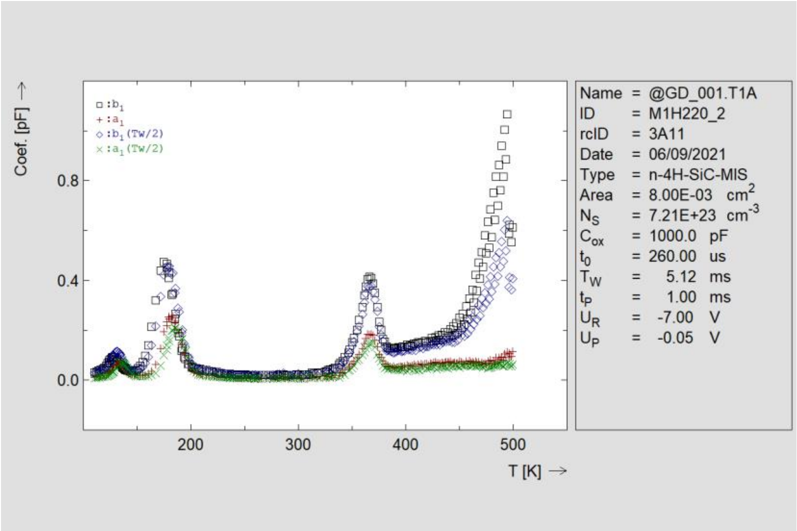

Measurements after 24 h of UIS stress

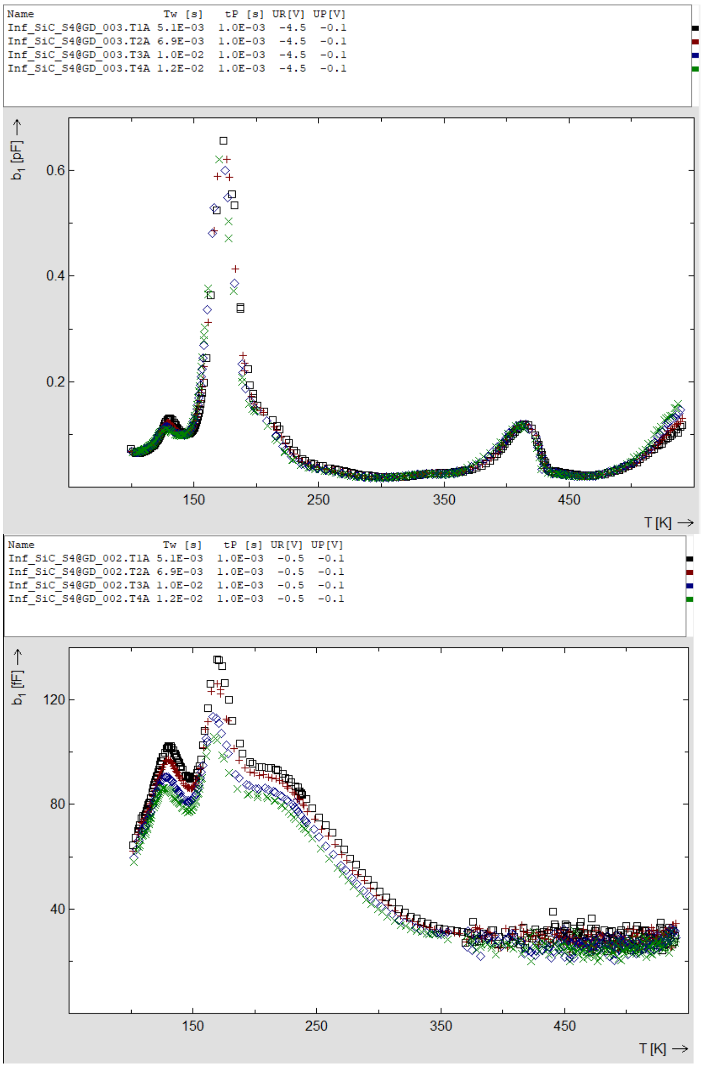

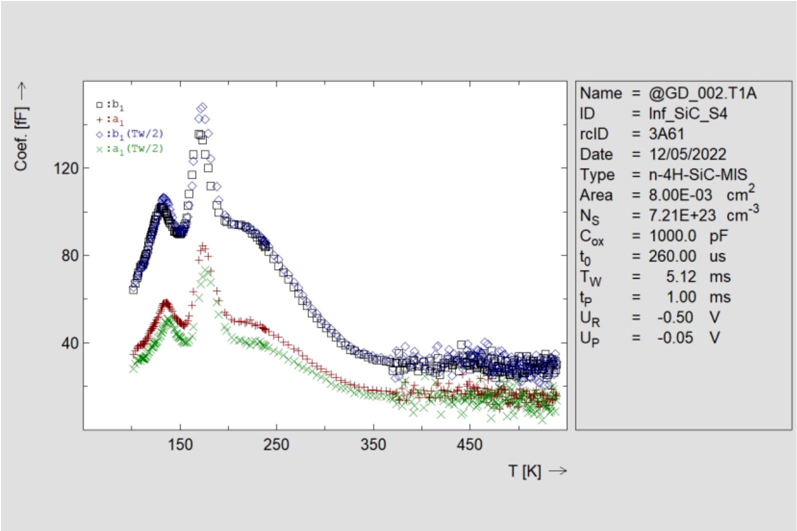

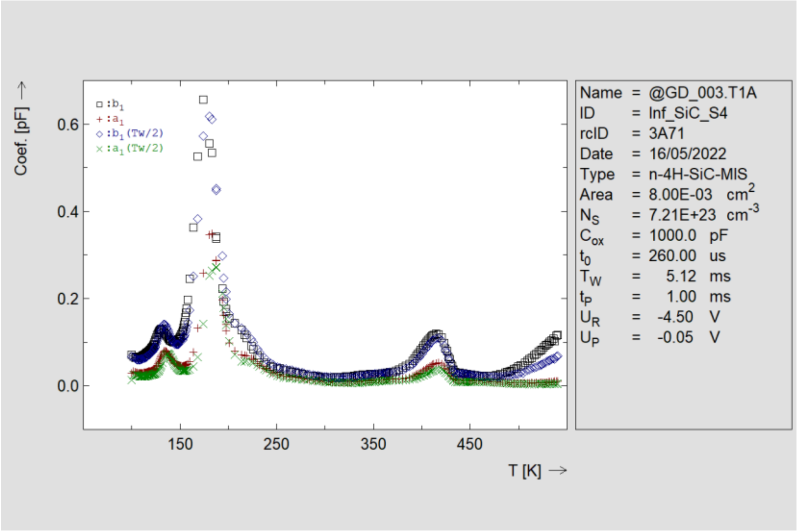

Static stress

Figure S1. Deep-level transient spectroscopy data.
